# Quality of Life in Children with Primary Antibody Deficiency

**DOI:** 10.1007/s10875-014-0072-x

**Published:** 2014-07-09

**Authors:** P. Titman, Z. Allwood, C. Gilmour, C. Malcolmson, C. Duran-Persson, C. Cale, G. Davies, H. Gaspar, A. Jones

**Affiliations:** 1 Immunology Department, Great Ormond Street Hospital, London, WC1N 3JH United Kingdom; 2 Institute of Child Health, University College London, London, UK UK

**Keywords:** Primary antibody deficiency, quality of life, psychological difficulty, immunoglobulin, children

## Abstract

Primary antibody deficiency disorders (PADs) can have an excellent outlook if diagnosed early and treated appropriately, but require lifelong treatment with immunoglobulin replacement. Some carry risks of inflammatory complications even with optimal treatment. Quality of life (QoL) and the psychological impact of PADs has been relatively little studied, particularly in children. The purpose of this study was to evaluate QoL and psychological impact in a large group of children affected by a range of PADs, as well as a group with transient hypogammaglobulinemia of infancy (THI). Both parental and, where appropriate, child ratings, were collected using standardised questionnaires (PedsQL and SDQ). Higher rates of psychological difficulties, particularly emotional and peer-relationship difficulties were found in children with PAD when compared with healthy controls. Quality of life was poorer than in healthy controls, and also worse than in children affected by diabetes mellitus. Variations in QoL and the degree of psychological difficulties were found between specific diagnostic groups, with children affected by THI being amongst those with the lowest scores for QoL. Further studies are needed to corroborate and extend these findings, but this study confirms previous findings that primary antibody deficiency has a significant impact on quality of life and psychological well-being, and additionally suggests that the impact varies according to severity of the underlying condition. For those with significant difficulties psychological intervention at an early stage may be beneficial.

**Electronic supplementary material:**

The online version of this article (doi:10.1007/s10875-014-0072-x) contains supplementary material, which is available to authorized users.

## Introduction

Primary antibody deficiency syndromes (PADs) account for the majority of primary immunodeficiency disorders [1]. Common Variable Immunodeficiency (CVID) occurs most frequently in the population, but in children single gene disorders are relatively more frequent, including X-linked agammaglobulinemia (XLA), hyper-IgM syndromes, rare autosomal recessive agammaglobulinemias, and a variety of less clearly defined disorders where antibody deficiency is the major defect. Some children who have undergone stem cell transplantation or gene therapy for severe forms of PID have incomplete immune reconstitution and have long-term antibody deficiency.

All forms of PAD carry a risk of long-term organ damage as a result of repeated or chronic infections. Some are also associated with other complications, including gastro-intestinal inflammatory disorders, autoimmune disease, liver disease, granulomatous disease, lymphoproliferative disease, and occasionally malignancy. However, with early diagnosis and optimal treatment the long-term outlook can be excellent [2].

The mainstay of treatment for PAD is immunoglobulin (antibody) replacement. Immunoglobulin can be administered intravenously (IVIg) every 3–4 weeks, or subcutaneously (SCIg) every 1–2 weeks. The aims of treatment are to maintain good health, prevent infections, prevent or arrest progression of complications, and maintain good quality of life. In recent years increased experience and more aggressive treatment with immunoglobulin, as well as better awareness and earlier diagnosis have led to improvements in the long-term outlook for physical health in patients with PADs [3]. However, the impact of PAD and its treatment on quality of life (QoL) has been relatively little studied. PADs carry potentially wide-ranging impacts psychologically, emotionally and practically for the whole family. Affected children (and their families) have to come to terms with a lifelong medical condition that requires treatment involving regular needles and considerable time input. In some disorders the long term outlook is more uncertain because of the risk of non-infective complications.

Children affected by transient hypogammaglobulinemia of infancy (THI) suffer from repeated infections in the first few years of life, and have low immunoglobulin levels that normalise over time [4]. Serious life-threatening infections are rare in this group, and immunoglobulin replacement is not usually indicated. Nevertheless their general health can be significantly affected. There is no diagnostic test to confirm THI, and the diagnosis can only be certain in retrospect, once immunoglobulin levels have normalized.

The aims of this study were to assess the impact on quality of life and on psychological, emotional and behavioural wellbeing in children affected by a range of PADs. The study included both child self-rating and parent-rating of the impact on the child’s QoL. Diagnostic categories of antibody deficiency were also compared to determine whether potentially more serious conditions have a greater impact..

## Methods

Children attending a specialist/tertiary immunology service with known or suspected antibody deficiency were invited to take part in a study investigating a range of aspects of PAD in children. Informed consent, and assent where appropriate, was obtained from all participants.

For the purposes of this study children were eligible if they were affected by a primary immunodeficiency requiring long-term immunoglobulin replacement, and in whom corrective treatment by stem cell transplantation or gene therapy was not indicated or, in the case of boys with CD40 ligand deficiency, no donor was available. In addition a small group of children with THI were studied.

## Participants

85 families were approached to take part in the wider study and 80 % consented to participate. Reasons for non-participation included families repeatedly not attending appointments, not having time to complete measures and perceiving that the study would involve extra visits to hospital. 68 children, 48 boys (70 %) and 20 girls (30 %) enrolled in the study. 43 children aged 4 years and above were eligible for the psychological difficulties and QoL study. These included 28 boys (65 %) and 15 girls (35 %), with a mean age of 10 years 8 months, range 5 – 16 years. One girl was withdrawn after the enrolment visit because the family no longer wished to participate.

37 children were receiving long-term immunoglobulin replacement, 35 subcutaneously, and 2 intravenously. Additionally, 1 child had received subcutaneous immunoglobulin for approximately 10 years and was able to discontinue during the course of the study because of normalisation of immunoglobulin levels. 5 further children with probable THI were not on replacement immunoglobulin.

Primary diagnosis is shown in Table I. For analysis of results by diagnosis the children with ‘CVID’ and ‘possible CVID’ have been amalgamated as ‘Combined’.

**Table 1:** PID diagnosis in study participants

Diagnosis	Number	Percent
Confirmed CVID^1^	13	30
Possible CVID^2^	3	7
Other PID^3^	9	21
X-Linked Agammaglobulinemia	5	12
Antibody deficiency post Bone Marrow Transplant for PID^4^	4	9
CD40 Ligand deficiency (no BMT) ^5^	3	7
(Probable) Transient hypogammaglobulinemia of Infancy (THI)	6	14
TOTAL	43	100

^1^According to European Society for Immunodeficiency (ESID) diagnostic criteria

^2^ Diagnosis not confirmed but suggestive according to ESID criteria

^3^ ICF syndrome (1), Activation-induced cytidine deaminase deficiency (autosomal recessive hyper-IgM deficiency) (1), Nijmegen breakage syndrome (1), Emanuel syndrome (chromosome 11/22 balanced translocation) with hypogammaglobulinemia (1), IPEX-like syndrome (1), undefined (4)

^4^ X-linked severe combined immunodeficiency (2), PNP deficiency (1), undefined (1)

^5^ No donor available for stem cell transplantation

## Measures

Standardised self-report questionnaires were used to assess psychological difficulties and quality of life.

Questionnaires were given to the parents and child (if appropriate) at the enrolment visit for the study; these visits were timed to coincide with routine follow-up out-patient clinic appointments, and did not take place on the same day as immunoglobulin infusions. Assistance was provided by the research nurse if necessary.

### Psychological Difficulties

Strengths and difficulties questionnaire (SDQ) [5].

This is a widely used standardised screening measure for common psychological difficulties in childhood and has been shown to have good reliability and validity. One parent of all children aged 4 – 16 completed the parent report version of the questionnaire, and children aged 11 or above completed the self-report version. This questionnaire measures 4 areas of psychological difficulties: conduct (behaviour) problems, emotional difficulties, hyperactivity, and peer relationship difficulties, which can be combined to give a total difficulties score. In addition, it includes a measure of prosocial behavior – this is a measure of positive behaviour and is not included in the total difficulties score. It also includes an impact score which measures the perceived impact of the child’s difficulties on their day to day life at home and at school. Children under the age of 4 were not included because younger children do not present with the types of psychological difficulties assessed by this measure.

### Quality of Life

Pediatric Quality of Life (PedsQL) [6, 7].

This is a widely used generic standardised measure of health related quality of life. It includes subscales measuring quality of life in four domains: physical, emotional, social and school. Parents of children aged 5 and above completed the parent report version of the questionnaire and children aged 5 and above completed the self-report version. The results from this study were compared with the normative values established in the UK standardization study of this measure [17], because these were considered to be the most appropriate comparison group, given the measure has not been used routinely for other patient groups within the hospital.

### Illness Severity

Severity of antibody deficiency can be assessed in various ways. These include the long term impact of the condition and the likelihood that it will result in serious health problems as an adult, the number and frequency of infections and use of antibiotics, and the impact of the illness on quality of life as perceived by the child or parent.

Since there is no standardized measure available for rating severity of an immunodeficiency, a 5 point clinician severity rating scale was developed for this study, with 5 indicating the most severe, and 1 the least severe conditions (see Online Resource 1). Severity ratings were based on clinician’s assessments of whether or not the condition was lifelong, and the presence of, or potential for, complications of the underlying PAD. The rating scale was shown to be reliable by assessing inter-rater reliability between a consultant immunologist and an experienced clinical nurse specialist (r = 0.83, *p* < 0.001).

Ethical approval for the study was granted by the Great Ormond Street Hospital/Institute of Child Health/UCL ethics committee.

## Results

### Psychological Outcome

**Fig. 1:**
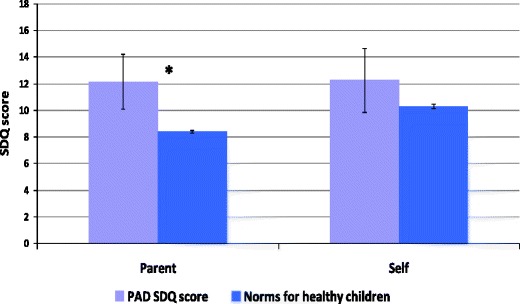
Mean total parent and child SDQ scores for psychological difficulties (including conduct, emotional, peer relationship and hyperactivity scores). Higher scores indicate more difficulties, with a maximum possible score of 40. Error bars indicate 95 % confidence intervals; and statistically significant differences are indicated by an asterisk *

**Fig. 2:**
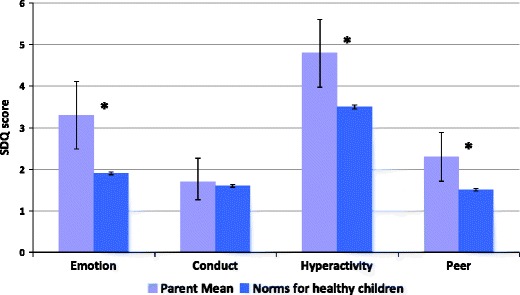
Mean parent rated SDQ individual subscale scores. Higher scores indicate higher levels of psychological difficulties with a maximum possible score of 10. Error bars indicate 95 % confidence intervals; statistically significant differences are indicated by an asterisk *

**Fig. 3:**
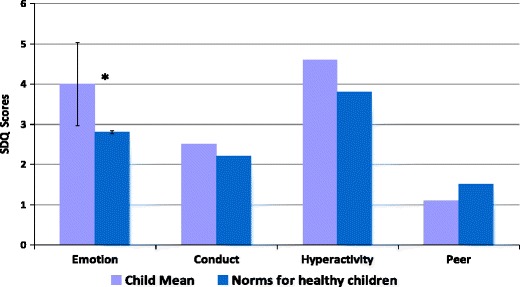
Mean child rated individual SDQ subscale scores. Higher scores indicate higher levels of psychological difficulty with a maximum possible score of 10.. Error bars are only shown for the emotional subscale; the intervals for other subscales are too large due to the small number of children who were old enough to complete this measure. Statistically significant differences are indicated by an asterisk *

43 parents and 19 children completed the SDQ. Figure 1 shows mean total SDQ scores for both parent and child reports for psychological difficulties. Figures 2 and 3 show mean parent and child SDQ score for the same four individual SDQ subscales. All scores were compared to normative values for the UK population. In order to focus more specifically on children with long-term antibody deficiency these analyses were also carried out excluding the THI group, but this did not affect the overall results, so data are presented for the whole cohort.

Parents of children with PAD reported significantly higher overall rates of psychological difficulties compared to normative values for healthy children. By subscale children with PAD had higher rates of emotional difficulties, peer relationship difficulties and hyperactivity, but no significant difference in behavioural (conduct) difficulties. There were no differences in ratings of prosocial behaviour (data not shown).

Children’s own ratings for total difficulties were higher than those of healthy children but this difference did not reach significance (*p* = 0.1). For individual subscale ratings children’s own scores for emotional difficulties were significantly higher than those for healthy children (*p* < 0.05).

Parent and child ratings on the SDQ were significantly correlated (r = 0.63, *p* < 0.01) indicating a high level of agreement on psychological difficulties.

### Quality of Life

43 parents and 39 children completed the PedsQL.

(i) Whole group

**Fig. 4:**
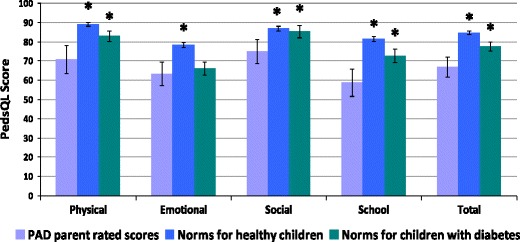
Mean parent rated overall quality of life and subscale scores for children with PAD, compared both with healthy children and with a group of children with diabetes mellitus included in the UK standardization study of the PedsQL [17]. Higher scores indicate better QoL and there is a maximum score of 100. Error bars indicate 95 % confidence intervals; statistically significant differences are indicated by an asterisk *

Figure 4 shows parent-rated quality of life for children with PAD compared with healthy children and also with children affected by diabetes mellitus reported in the UK standardisation study for the PedsQL (Upton et al. 2005). These children were chosen as an illness comparison group because diabetes mellitus is also a lifelong condition that requires regular medication, including frequent needles and blood tests.

**Fig. 5:**
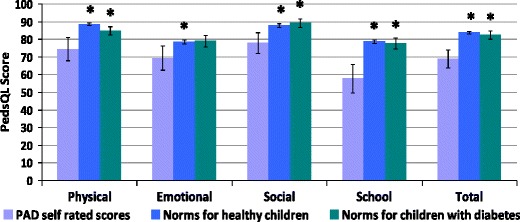
Mean child rated overall quality of life and subscale scores compared both with healthy children and with a group of children with diabetes mellitus included in the UK standardization study of the PedsQL [17]. Higher scores indicate better QoL and there is a maximum score of 100.. Error bars indicate 95 % confidence intervals; statistically significant differences are indicated by an asterisk *

Children’s own ratings of overall quality of life and subscale scores are shown in Fig. 5, also compared with healthy children and children with diabetes.

Parents of children with PAD reported significantly lower quality of life in their children on all the subscales of the PedsQL when compared with healthy UK children. In addition, parents’ ratings were lower than parents of children with diabetes for all subscales, with the exception of the emotional subscale.

A similar pattern was observed for child ratings of QoL; children with PAD reported significantly lower QoL compared to healthy children on all subscales (including the overall score) of the PedsQL, and lower scores than children with diabetes on all subscales except the emotional subscale.

Parent and child ratings on PedsQL were very strongly correlated (r = 0.79) indicating high levels of agreement on quality of life between parents and children.

(ii) By diagnostic group

**Fig. 6:**
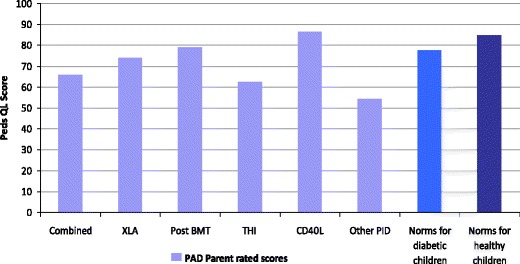
Mean parent rated overall quality of life by diagnostic group Higher scores indicate better QoL and there is a maximum score of 100. For analysis of results by diagnosis the children with ‘CVID’ and ‘possible CVID’ have been amalgamated as ‘Combined’

**Fig. 7:**
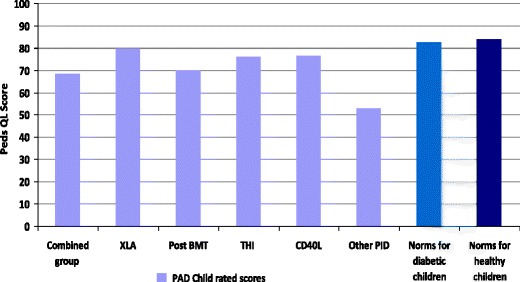
Mean child rated overall quality of life by diagnostic group Higher scores indicate better QoL and there is a maximum score of 100. For analysis of results by diagnosis the children with ‘CVID’ and ‘possible CVID’ have been amalgamated as ‘Combined’

Figures 6 and 7 show the mean overall QoL rating for parents and children respectively, by diagnostic group. The numbers of children in each group were small, so no statistical analysis was possible, and results should therefore be interpreted with caution. The ‘Other PID’ group reported the lowest quality of life scores.

### Severity of Illness

**Fig. 8:**
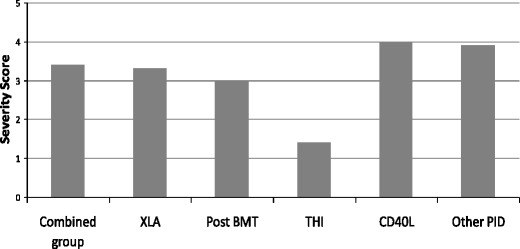
Mean clinician severity ratings for each of the diagnostic groups. The maximum score is 5, indicating most severe. For analysis of results by diagnosis the children with ‘CVID’ and ‘possible CVID’ have been amalgamated as ‘Combined’

Mean clinician severity ratings for each of the diagnostic groups is shown in Fig. 8. Comparison of these scores with the parent and child reported QoL and parental reports of psychological difficulties reveals very low correlation (parent or child report of quality of life: r =–0.09 and r =–0.26 respectively; parental report of psychological difficulties: r = 0.19) In particular, whilst the clinician severity rating for THI was the lowest of all the conditions, both children and parents in this group reported relatively poor QoL and high rates of psychological difficulties, second only in severity to the ‘Other PID’ group.

## Discussion

This current study shows that children with a range of primary antibody deficiencies have higher rates of psychological difficulties compared to healthy children. This is consistent with findings in many other types of chronic illness in childhood, where children have also been shown to be at higher risk of psychological disorders [8]. Specifically, children in this study had higher rates of emotional difficulties, peer relationship difficulties and hyperactivity, but did not have higher rates of behavioural (conduct) disorders. These differences are clinically meaningful and equivalent to moderate to large effect sizes, representing the highest (most affected) 20 % of children in the general population in terms of levels of psychological difficulties. This pattern of psychological difficulties is in contrast to studies of children in the general population, where the most common types of difficulty children experience are conduct or behavioural problems. However, it is consistent with studies in children with other types of primary immunodeficiencies [9, 10], which have also found higher rates of emotional and or peer relationship difficulties in affected children. At present there are no intervention studies specifically with children with these types of medical conditions. However, it is likely that treatments based on family oriented cognitive behavioural principles, which have been found to be effective with unaffected children with these types of psychological difficulties may be appropriate, but further research is needed on evaluating treatment approaches

Both children and parents reported lower levels of QoL than healthy children, but, perhaps more surprisingly, they also reported lower QoL than a comparison group of children with diabetes mellitus. This comparison group was chosen because it has similar implications to PAD, being a lifelong disorder, requiring treatment with regular needles, and carrying significant risks of life-threatening and life-limiting complications. The finding of worse QoL in children with PAD than those with diabetes is unexpected in light of the belief amongst clinicians that QoL can be ‘normal’ in children with PADs provided that treatment is optimised. A noticeable finding is that scores relating to school were particularly low; which might reflect a sense of isolation resulting from fear of infections or of difference from peers. The data for the comparison group of children with diabetes was taken from the UK standardization of the quality of life measure [7] but in future research it would be important to collect data on a matched control group if possible.

A possible confounding factor is that timing of completion of questionnaires in relation to immunoglobulin infusions could have had an impact on responses, particularly if there were associated adverse reactions. However, none of the children received their infusions on the day of completing questionnaires, and there were no significant adverse reactions in any of the participants during the course of the study. All children were well-established and stable on long-term treatment

A small number of previous studies have investigated QoL in PADs. Several have focussed on comparison between IVIg and SCIg, demonstrating broadly improved QoL in both adults and children who switch from IV to SC treatment, particularly when treatment is given at home [11–13]. Other studies have compared QoL in adults and children affected by PADs with healthy controls and other chronic diseases. These suggest that QoL in adults with CVID is worse than in healthy controls and patients with congestive heart failure or diabetes mellitus [14, 15], but QoL in adult males with XLA is similar to healthy controls [16, 17].

Whilst most other studies of children with PIDs have also shown a significant impact on QoL when compared to healthy controls, results have varied when compared to other health conditions. Zebracki et al. [18] found that parents reported lower QoL in children with various PIDs (all requiring IVIg) than in healthy controls, but similar to children with juvenile arthritis. Mozaffari et al. [19] found self-reported QoL in children with PIDs was lower than for healthy aged-matched controls. A study of children and adolescents affected by X-linked agammaglobulinemia, which included both parental and self-reporting, showed poorer QoL in affected boys than in healthy controls, but better than in children with rheumatic disorders [20]. These paediatric studies have been performed in populations from diverse geographical backgrounds, and the availability of early diagnosis and access to optimal treatment may vary, but the findings show consistently that quality of life in children with PID is worse in all groups than in healthy controls. Future research should focus on determining what factors are related to variation in QoL, such as the impact of the effectiveness of treatment in improving QoL.

A range of antibody deficiency disorders were included in the study. Variations in QoL were observed between diagnostic groups but the small numbers in each group did not permit assessment of significance. At the moment, these results must be interpreted with caution given the small numbers of participants in this sample, however, this is an important area to study in future and may explain variations in impact of PADs on QOL seen in previous studies. For example, boys affected by XLA and children in the post-BMT group reported higher levels of QoL, consistent with the low incidence of non-infectious complications in these two groups. Children with CVID and ‘other’ PAD reported lower QoL, consistent with the higher incidence of other complications they experience. However, surprisingly, for the small group of boys with CD40 ligand deficiency (which carries a high risk of liver disease and other complications) parental QoL ratings were higher than for all other groups. Conversely, parent reports for the group with ‘probable THI’ reported lower QoL than all groups except 'other PAD'. THI is not associated with serious infections or complications, and affected children are usually managed with antibiotics. They are monitored with regular blood tests to ensure that immunoglobulin levels are improving, and reassurance to parents that the likely outcome will be normality. However, these findings suggest that the impact of THI on children and families is greater than expected, and may raise questions about management of such children. It is also possible that this reflects the relatively short time that the parents have had to adjust to their child’s condition, since the children with THI were somewhat younger and therefore there will have been a shorter time since diagnosis. Additionally the uncertainty about the long-term outlook may contribute to the findings. Further studies are needed in this group to determine if this result is replicated and to establish whether the impact is significant enough in some cases to justify more aggressive treatment – in particular whether more of these children might benefit from short to medium-term immunoglobulin replacement.

There have been no previous attempts to correlate QoL with severity of underlying disorders. Objective ratings of severity of illness by clinicians revealed discrepancies with the child and parents’ ratings of the impact on QoL. According to clinician ratings, CD40 ligand deficiency is the ‘most severe’ of the PAD disorders in this study, while XLA, CVID, other PAD, and post-BMT PAD are rated as intermediate, and THI is significantly milder. These perceptions do not tally with the QoL data, and this illustrates the importance of considering patient/parent reported outcomes, as well as more ‘objective’ ratings based on clinical experience.

When the study was established it was hoped that comparisons could be made between children receiving IVIg and SCIg. However, the majority of children on long-term immunoglobulin at this centre are on SCIG, only 2 children in this cohort receiving their immunoglobulin intravenously. It was therefore not possible to compare the two groups in terms of QoL or psychological difficulties. Previous research has demonstrated improved QoL for both children and adults when moving from IVIG to SCIG, with emphasis on the impact of home versus hospital-based therapy.

There are limitations to this study which mean the results do need to be seen as preliminary; future research should address these if possible. Firstly, the results from the PAD group were compared with normative values for the standardized measures rather than with a matched control group of children. Future research should include an appropriate control group, for example contemporaneous controls with other medical conditions treated at the same hospital. In addition this was a cross sectional study so it was not possible to relate results to the child’s medical treatment, and in particular whether infections were well controlled and if they were receiving optimal treatment. Although the overall numbers of patients was relatively high compared to other studies of children with these rare conditions, the numbers in individual diagnostic groups and age groups were relatively small, limiting the comparisons that could be made. Future studies should ideally have enough power to investigate these factors, which may have an important impact on both psychological outcome and QoL but could not be included in this study.

## Conclusions

This study confirms previous findings that primary antibody deficiency has a significant impact on quality of life and psychological well-being, and additionally suggests that the impact varies according to severity of the underlying condition. For children with significant difficulties psychological intervention at an early stage may be beneficial.

## Electronic supplementary material

Below is the link to the electronic supplementary material.

ESM 1 (PDF 90 kb)
