# Supplementary material for: Quality of Life in Children with Primary Antibody Deficiency
Source: J Clin Immunol. 2014 Jul 9;34(7):844–52. doi: 10.1007/s10875-014-0072-x (PMC4165866; doi:10.1007/s10875-014-0072-x)
Supplement: Supplementary file 1 — (PDF 90 kb) [file 10875_2014_72_MOESM1_ESM.pdf]

Online resource 1

Quality of Life in Children with Primary Antibody Deficiency

Journal of Clinical Immunology

Titman P[1], Allwood Z[1], Gilmour C[1], Malcolmson C[1], Duran-Persson C[1], Cale C[1], Davies G[1], Gaspar H[2], Jones A[1]
[1] Great Ormond Street Hospital for Children, London
[2] Institute of Child Health, University College London

Corresponding author email: alison.jones@gosh.nhs.uk

Caption: **Illness severity scale for children affected by Primary Antibody Deficiency disorders**

Severity score	Duration of disorder	Complications
1	Not lifelong	No
2	Not lifelong	Yes
3	Lifelong	No
4	Lifelong	Early
5	Lifelong	Established
